# Lipoprotein (a) and myocardial infarction: impact on long-term mortality

**DOI:** 10.1186/s12944-023-01841-z

**Published:** 2023-06-09

**Authors:** Jian Zhang, Lin Jia, Yu Yang, Ai Xiao, Xianhe Lin

**Affiliations:** Cardiology Department, The First Affiliated Hospital of Anhui Medical University, Hefei, Anhui China

**Keywords:** Myocardial infarction, Lipoprotein (a), Left ventricular ejection fraction

## Abstract

**Background and aims:**

Lipoprotein (a) [Lp(a)] is a genetically regulated lipoprotein particle that is an independent risk factor for coronary atherosclerotic heart disease. However, the correlation between Lp(a) and left ventricular ejection fraction (LVEF) in patients with myocardial infarction (MI) has been poorly studied. The present study investigated the correlation between Lp(a) and LVEF, as well as the impact of Lp(a) on long-term mortality in patients with MI.

**Methods:**

Patients who underwent coronary angiography resulting in MI diagnosis between May 2018 and March 2020 at the First Affiliated Hospital of Anhui Medical University were included in this study. The patients were divided into groups based on the Lp(a) concentration and LVEF (reduced ejection fraction group: < 50%; normal ejection fraction group: ≥ 50%). Then, correlations between the Lp(a) level and LVEF, as well as the impact of Lp(a) on mortality, were assessed.

**Results:**

This study included 436 patients with MI. The Lp(a) level and LVEF were significantly and negatively correlated (r = -0.407, β = -0.349, *P* < 0.001). The area under the receiver operating characteristic curve (ROC) indicated that an Lp(a) concentration > 455 mg/L was the best predictive value for reduced ejection fraction (AUC: 0.7694, *P* < 0.0001). The clinical endpoints did not differ based on the Lp(a) concentration. However, all-cause mortality and cardiac mortality differed based on LVEF.

**Conclusions:**

These results suggest that an elevated Lp(a) concentration predicts reduced ejection fraction and that LVEF predicts all-cause mortality and cardiac mortality in patients with MI.

**Supplementary Information:**

The online version contains supplementary material available at 10.1186/s12944-023-01841-z.

## Introduction

Coronary atherosclerotic heart disease and its complications are one of the leading causes of mortality and disability in the global population [1, 2]. Myocardial infarction (MI) is one of the most severe types, manifested by a dramatic reduction in coronary blood flow and a severe imbalance between oxygen supply and demand, which often requires myocardial reperfusion therapy (including thrombolytic therapy, percutaneous coronary intervention (PCI) and coronary artery bypass graft (CABG)) to restore myocardial perfusion. However, the processes of ischemia and reperfusion may lead to myocardial cell damage or necrosis, affecting cardiac pumping and the development of heart failure, which imposes a serious burden on society and individuals [3, 4]. It is well known that hyperlipidemia is an independent risk factor for coronary atherosclerosis [5, 6]. It aggravates the complexity and severity of coronary artery lesions and affects the long-term prognosis of patients. Lp(a) is a separate lipoprotein species that is mainly regulated by genetic genes. Lp(a) is composed of cholesterol-rich low-density lipoproteins [1]. The concentration of Lp(a) in plasma is mainly determined by the LPA gene [7]. In the last decade, data from epidemiological studies and meta-analyses [8], Mendelian randomization studies [9] and genome-wide association studies [10, 11] have ultimately demonstrated that elevated Lp(a) levels lead to a higher risk of cardiovascular disease in the population, mainly including but not limited to myocardial infarction, stroke, and peripheral arterial disease [12]. The higher the concentration of Lp(a) is, the more severe the degree of coronary artery lesions (assessed by the SYNTAX score or Gensini score) [1, 13, 14]. Most recently, the EAS/ESC guidelines recommended that all individuals should have Lp(a) measured at least once [15]. However, the prognostic impact of Lp(a) is still controversial [13, 16, 17].

Thus, this study investigated the correlation between the Lp(a) level and LVEF and the impact of Lp(a) on long-term mortality in patients with MI to clarify this relationship.

### Patients and methods

Study population: This was a single-center, observational cohort study. MI, including non-ST-segment elevation myocardial infarction (NSTEMI) and ST-elevation myocardial infarction (STEMI), was defined as chest pain with new ST-segment changes and elevation of myocardial necrosis markers to at least twice the upper limit of the normal range. Inclusion criteria: A total of 472 consecutive patients who underwent coronary angiography and were diagnosed with MI at the Department of Cardiovascular, First Affiliated Hospital of Anhui Medical University between May 2018 and March 2020. Of the 472 patients, 36 patients were excluded according to the exclusion criteria, which were (1) incomplete clinical data (*n* = 13), (2) previous coronary artery bypass grafting (CABG) (*n* = 5), (3) malignancies (*n* = 7), and (4) loss to follow-up (*n* = 11). Finally, 436 patients were included in this analysis.

The institutional ethics committee of The First Affiliated Hospital of Anhui Medical University approved this study, which complied with the Declaration of Helsinki. All patients provided written informed consent to participate, and all information related to the patients’ identities was concealed.

### Definition of risk factors

The choice of variables mainly includes risk factors for coronary heart disease, prognostic indicators of myocardial infarction, details on myocardial infarction, and treatment of myocardial infarction. Body mass index (BMI) was calculated as follows: BMI = weight (kg)/height^2^ (m^2^). Pulse pressure was calculated as follows: Pulse pressure (mmHg) = systolic blood pressure (mmHg)—diastolic blood pressure (mmHg). Hypertension was diagnosed based on either of the following criteria: (1) ongoing antihypertensive therapy and (2) three blood pressure measurements at rest with a systolic blood pressure ≥ 140 mmHg or diastolic blood pressure (DBP) ≥ 90 mmHg. Diabetes mellitus (DM) was diagnosed based on any of the following criteria: (1) a definite diagnosis by a physician, (2) current long-term use of diabetes-related medications, and (3) a fasting blood glucose level of ≥ 7.0 mmol/L, a 2 h postprandial blood glucose level of ≥ 11.1 mmol/L, or a random blood glucose level of ≥ 11.1 mmol/L by oral glucose tolerance test. A history of stroke, percutaneous coronary intervention (PCI), and MI were derived from information provided by the patient and then confirmed by relevant laboratory tests. The neutrophil-to-lymphocyte ratio (NLR) was calculated as follows: NLR = neutrophil (*10^9^/L)/lymphocyte (*10^9^/L).

### Data collection

Venous blood was collected after the second day of hospitalization (fasting > 8 h). Routine blood tests, triglycerides (TG), total cholesterol (TC), low-density lipoprotein cholesterol (LDLC), high-density lipoprotein cholesterol (HDL-C), very low-density lipoprotein cholesterol (VLDL-C), apolipoprotein A-I (ApoA-I), apolipoprotein B (ApoB), Lp(a), glomerular filtration rate (eGFR), uric acid, and fasting blood glucose (FBG) levels were measured by standard laboratory methods. The concentrations of plasma TG, TC, LDL-C, HDL-C, VLDL-C, ApoA-I, ApoB and Lp(a) were measured using an automatic biochemistry analyzer (Hitachi 7150, Tokyo, Japan) and assayed by an immunoturbidimetry method according to the manufacturer’s instructions. The left ventricular ejection fraction (LVEF) was calculated using the Simpson method. The Simpson method is not limited by a fixed geometric pattern and is suitable for patients with coronary artery disease with ventricular wall segmental motion, but the measurement and calculation methods are complex and usually processed by computer analysis. The number of cross-sections should be increased as much as possible when the left ventricular morphology changes, and accurate results can be obtained by computer processing or by 3D ultrasound. Vital signs, past medical history, smoking, drinking, laboratory tests, and electrocardiogram data, among others, were extracted from the electronic medical record management system of the First Affiliated Hospital of Anhui Medical University.

### Clinical endpoint events

Professional staff followed up with the patients through clinical visits or telephone contact. All patients were followed up until March 16, 2023, with a median follow-up time of 48 (IQR: 45, 53) months. The clinical endpoint events included all-cause mortality and cardiac mortality. All-cause mortality was defined as death attributable to cardiac or noncardiac causes. Cardiac mortality was defined as death due to MI, heart failure, sudden cardiac death, or cardiac surgery.

### Statistical analyses

SPSS 26.0.0 (IBM Corp., Armonk, NY, USA) and GraphPad Prism 9.0.2 (GraphPad Software, San Diego, CA, USA) were used for the statistical analyses and to create graphs, respectively. Method to test whether data obey normal distribution: normality test (Kolmogorov‒Smirnov test. Continuous variables were reported as the means ± standard deviations or medians (interquartile ranges) depending on the normal distribution test. An analysis of variance was used to assess between-group differences for normally distributed continuous variables, and the Kruskal–Wallis H test was used for nonnormally distributed continuous variables. Categorical variables were reported as numbers (percentages); the chi-squared or Fisher’s exact tests were used to assess between-group differences. Spearman’s correlation coefficient was used to evaluate correlations between each independent variable and the LVEF. A plot of the correlation between the Lp(a) concentration and the LVEF was created, and variables with a *P* value of < 0.1 were included in the multivariate linear regression analysis.

The patients were divided into two groups (reduced ejection fraction group; normal ejection fraction group) based on an LVEF cutoff value of 50%. Significant independent variables for predicting the normal ejection fraction group (*P* < 0.1) were identified by univariate logistic regression analysis; significant factors were included in the multivariate logistic regression analysis using the stepwise forward method. Receiver operating characteristic (ROC) curves were generated to analyze the best predictive value of Lp(a) for predicting normal ejection fraction, which included sensitivity and specificity calculations.

Clinical endpoint event predictions based on different independent variables were investigated by univariate Cox regression analysis; independent variables with a *P* value of < 0.1 were included in the multivariate Cox regression analysis. Finally, ROC curves were used to assess the reliability of Lp(a) and LVEF for predicting mortality, and Kaplan–Maier curves were used to illustrate the risk of mortality in the reduced and normal EF groups.

## Results

### Baseline clinical characteristics

This study included 436 patients; the median age was 65 years, 323 patients (71.4%) were male, and the median follow-up time was 48 months. Table 1 presents the study population’s baseline clinical characteristics.

**Table 1:** Baseline characteristics of patients by tertiles of lipoprotein (a)

**Variables**	**Lp(a)(mg/L)**				**P Value**
	**Tertile 1**	**Tertile 2**	**Tertile 3**	**Total**	
	**(*n* = 145)(49–201)**	**(*n* = 146)(202–338)**	**(*n* = 145)(342–1200)**	**(*n* = 436)(49–1200)**	
**Hospitalization time (days)**	11 (8–14)	10 (7–14)	11 (8–15)	11 (8–14)	0.343
**Age (years)**	62 (52–72)	64 (54–74)	68 (60–76)	65 (54–74)	< 0.001
**Male**	108 (74.5%)	113 (77.4%)	102 (70.3%)	323 (74.1%)	0.386
**BMI (kg/m**^**2**^**)**	24.36 ± 3.36	24.21 ± 3.60	24.12 ± 3.46	24.23 ± 3.47	0.838
**Heart rate (beat/min)**	72 (65–82)	76 (68–86)	78 (70–89)	76 (68–86)	0.005
**Pulse pressure (mmHg)**	52 (45–60)	49 (40–58)	50 (40–60)	50 (41–59)	0.078
**Current smoker**	53 (36.6%)	54 (37.0%)	40 (27.6%)	147 (33.7%)	0.161
**Current drinker**	35 (24.1%)	46 (31.5%)	26 (17.9%)	107 (24.5%)	0.027
**Hypertension**	73 (50.3%)	75 (51.4%)	81 (55.9%)	229 (52.5%)	0.606
**Diabetes mellitus**	28 (19.3%)	16 (11.0%)	28 (19.3%)	72 (16.5%)	0.086
**Previous stroke**	12 (8.3%)	17 (11.6%)	22 (15.2%)	51 (11.7%)	0.188
**Previous PCI**	5 (3.4%)	7 (4.8%)	8 (5.5%)	20 (4.6%)	0.694
**Previous MI**	2 (1.4%)	4 (2.7%)	7 (4.8%)	13 (3.0%)	0.215
**LVEF (%)**	59 (55–62)	58 (54–60)	54 (49–57)	57 (53–59)	< 0.001
**NLR**	3.99 (2.46–6.46)	4.42 (2.76–7.54)	4.19 (2.82–7.47)	4.20 (2.69–7.09)	0.218
Monocyte (*10^**9**^**/L)**	0.49 (0.36–0.68)	0.46 (0.36–0.67)	0.48 (0.36–0.60)	0.48 (0.36–0.64)	0.813
**Hemoglobin (g/L)**	137 ± 21	134 ± 20	130 ± 20	134 ± 21	0.022
Platelet (*10^**9**^**/L)**	205 (162–236)	198 (157–244)	192 (159–240)	200 (161–239)	0.868
**TG (mmol/L)**	1.37 (1.05–1.99)	1.43 (1.06–1.94)	1.32 (0.98–1.65)	1.37 (1.02–1.86)	0.086
**TC (mmol/L)**	3.98 (3.41–4.72)	4.29 (3.68–4.97)	4.03 (3.38–4.74)	4.08 (3.46–4.88)	0.202
**LDL-C (mmol/L)**	2.42 (1.96–3.00)	2.65 (2.18–3.11)	2.50 (1.99–3.07)	2.53 (2.01–3.09)	0.210
**HDL-C (mmol/L)**	1.01 (0.84–1.19)	1.03 (0.86–1.15)	1.03 (0.89–1.18)	1.02 (0.86–1.18)	0.953
**VLDL-C (mmol/L)**	0.51 (0.38–0.70)	0.53 (0.40–0.69)	0.48 (0.36–0.61)	0.51 (0.37–0.66)	0.088
**ApoA-I (g/L)**	1.07 (0.94–1.22)	1.09 (0.91–1.20)	1.06 (0.94–1.22)	1.07 (0.92–1.21)	0.889
**ApoB (g/L)**	0.79 (0.69–0.91)	0.82 (0.70–0.98)	0.76 (0.68–0.98)	0.79 (0.69–0.96)	0.367
**Lp(a) (mg/L)**	149 (120–178)	264 (235–293)	480 (412–612)	264 (178–412)	< 0.001
eGFR (ml/(min*1.73m^**2**^**))**	100 (90–113)	97 (86–109)	89 (73–104)	96 (82–109)	< 0.001
**Uric acid (umol/L)**	340 (280–393)	366 (313–423)	403 (320–459)	366 (305–432)	< 0.001
**FBG (mmol/L)**	5.83 (5.23–6.93)	6.05 (5.31–7.14)	6.50 (5.45–7.89)	6.08 (5.31–7.34)	0.007
**NSTEMI**	72 (49.7%)	77 (52.7%)	71 (49.0%)	220 (50.5%)	0.790
**STEMI**	73 (50.3%)	69 (47.3%)	74 (51.0%)	216 (49.5%)	
**Number of occluded arteries**	102 (70.3%)	100 (68.5%)	93 (64.1%)	295 (67.7%)	0.510
**PCI/CABG**	112 (77.2%)	104 (71.2%)	96 (66.2%)	312 (71.6%)	0.114
**Antiplatelet agent**	139 (95.9%)	127 (87.0%)	132 (91.0%)	398 (91.3%)	0.133
**ACEI/ARB**	73 (50.3%)	62 (42.5%)	56 (38.6%)	191 (43.8%)	0.302
**Beta-blocker**	75 (51.7%)	88 (60.3%)	83 (57.2%)	246 (56.4%)	0.046
**CCB**	16 (11.0%)	19 (13.0%)	19 (13.1%)	54 (12.4%)	0.675
**Diuretic**	17 (11.7%)	18 (12.3%)	37 (25.5%)	72 (16.5%)	0.001
**Statins**	137 (94.5%)	129 (88.4%)	127 (87.6%)	393 (90.1%)	0.374

Values are expressed as the mean ± standard deviation or median (interquartile range), n (%). *P* values were calculated using ANOVA, Kruskal‒Wallis test, chi-square test or Fisher's test. *P* < 0.05 indicated statistical significance

The patients were divided into three groups based on the ladder Lp(a) concentration. Lipid levels, such as TG, TC, LDL-C, HDL-C, VLDL-C, ApoA-I, and ApoB, did not differ among the groups. Uric acid significantly differed only between Tertile 1 and Tertile 2. Age, heart rate, LVEF, hemoglobin, eGFR, uric acid, and FBG significantly differed only between Tertile 1 and Tertile 3. Age, current alcohol consumption, LVEF, and eGFR significantly differed only between Tertile 2 and Tertile 3. The remaining indicators, such as hospitalization time, sex, BMI, pulse pressure, current smoking status, hypertension, DM, previous stroke, previous PCI, previous MI, NLR, monocytes, and platelets, did not differ among the groups. Details about MI (STEMI, NSTEMI, number of occluded arteries) did not differ among the groups. For the treatment of myocardial infarction, PCI or CABG, antiplatelet agents, ACEIs/ARBs, CCBs, and statins also did not differ between groups. However, beta-blockers were significantly different between Tertile 1 and Tertile 2. Diuretics differed significantly between Tertile 1 and Tertile 3 and between Tertile 2 and Tertile 3.

### Lp(a) levels and LVEF

**Fig. 1:**
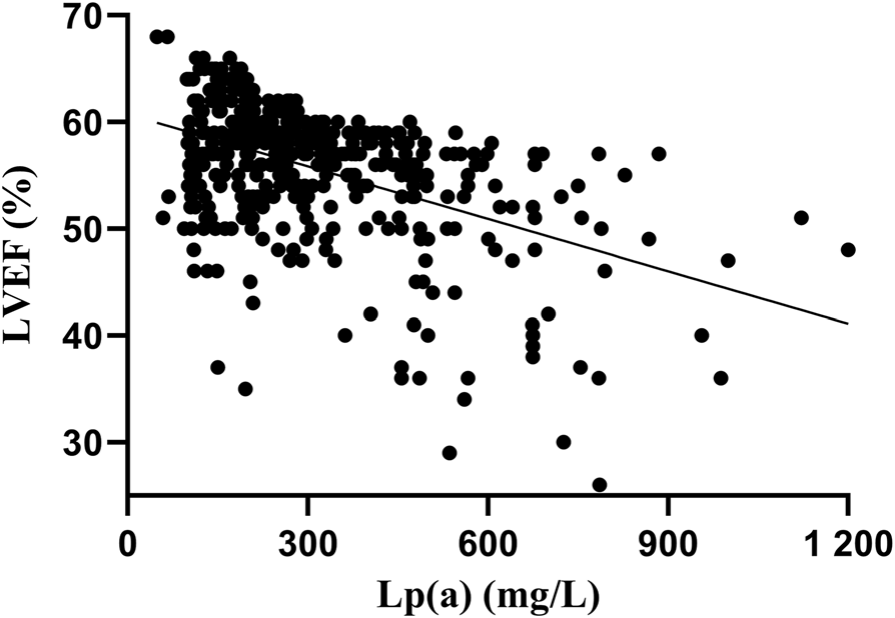
Scatter diagram showing the correlation between Lp(a) levels and LVEF. Lp(**a**) and LVEF had a strong and significant correlation (r = -0.407, *P* < 0.001)

Figure 1 presents a scatter diagram of the Lp(a) concentrations and LVEF; the correlation was significantly and negatively correlated (r = -0.407, *P* < 0.001). The univariate linear regression showed that age, heart rate, pulse pressure, DM, previous stroke, NLR, monocytes, hemoglobin, ApoA-I, eGFR, uric acid, FBG and Lp(a) levels were significantly and independently associated with LVEF (all *P* < 0.05). These variables were included in the multivariate linear regression analysis, and age, heart rate, pulse pressure, eGFR, FBG and Lp(a) levels remained significantly and independently associated with LVEF (all *P* < 0.05). Furthermore, Lp(a) remained significantly and negatively associated with LVEF (standardized coefficient β = -0.349, *P* < 0.001; Table 2).

**Table 2:** Multivariate linear regression analysis of LVEF

**Variables**	**Unstandardized coefficients(B)**	**Standardized coefficients(β)**	**t**	*P value*	**F**	**VIF**
**Age**	-0.131	-0.244	-5.422	< 0.001	62.894	1.632
**Heart rate**	-0.048	-0.123	-3.375	0.001		1.071
**Pulse pressure**	0.033	0.077	2.138	0.003		1.040
**Lp(a)**	-0.012	-0.349	-9.504	< 0.001		1.089
**eGFR**	0.045	0.150	3.260	0.001		1.702
**FBG**	-0.721	-0.248	-6.850	< 0.001		1.059

**Fig. 2:**
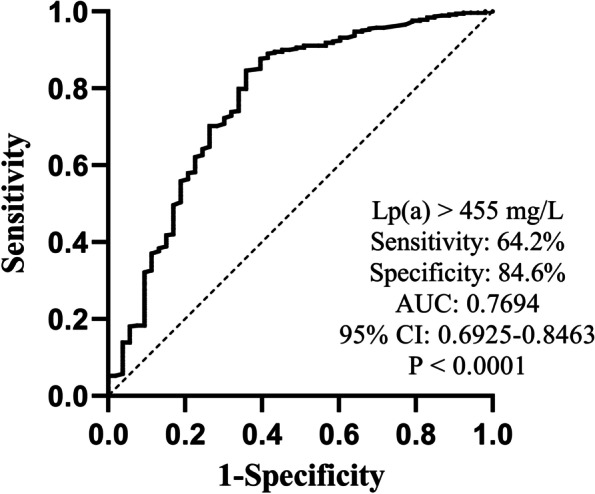
ROC curve analysis of Lp(a) for reduced LVEF. The Lp(**a**) cutoff value of 455 mg/L on admission predicts a reduced LVEF in patients, with a sensitivity of 64.2% and a specificity of 84.6%. The AUC was 0.7694 (95% CI 0.6925 to 0.8463; *P* < 0.0001)

Univariate logistic regression was used to analyze the predictive value of each independent variable for normal LVEF (LVEF ≥ 50%). Hospitalization time, age, male sex, heart rate, DM, hemoglobin, ApoA-I, eGFR, uric acid, FBG and Lp(a) levels were significantly and independently associated with the normal LVEF group (all *P* < 0.05). Therefore, these variables were included in a multivariate logistic regression analysis, and ApoA-I (odds ratio [OR]: 6.189; 95% confidence interval [CI]: 1.169–32.763; P = 0.032), Lp(a) level (OR: 0.996; 95% CI: 0.994–0.997; *P* < 0.001), eGFR (OR: 1.053; 95% CI: 1.035–1.071; *P* < 0.001), and FBG (OR: 0.825, 95% CI: 0.730–0.933, *P* = 0.002) remained significantly associated with the normal LVEF group (Table 3). Based on the ROC curve, an Lp(a) concentration of 455 mg/L had the best predictive value for reduced LVEF (area under the curve (AUC): 0.7694; 95% CI: 0.6925–0.8463; sensitivity: 64.2%; specificity: 84.6%; *P* < 0.0001; Fig. 2).

**Table 3:** Univariate and multivariate logistic regression analysis of normal LVEF

**Variables**	**Univariate logistic regression analysis**			**Multivariate logistic regression analysis**		
	**OR**	**95% CI**	***P* value**	**OR**	**95% CI**	***P* value**
**Hospitalization time (days)**	0.929	0.881–0.979	0.006	#	#	0.149
**Age (years)**	0.929	0.903–0.956	< 0.001	#	#	0.145
**Male**	1.890	1.035–3.452	0.038	#	#	0.281
**BMI (kg/m**^**2**^**)**	1.028	0.945–1.118	0.519			
**Heart rate (beat/min)**	0.974	0.959–0.990	0.001	#	#	0.254
**Pulse pressure (mmHg)**	1.009	0.989–1.029	0.391			
**Current smoker**	1.656	0.856–3.204	0.134			
**Current drinker**	1.458	0.706–3.013	0.308			
**Hypertension**	0.695	0.387–1.249	0.224			
**Diabetes mellitus**	0.443	0.229–0.858	0.016	#	#	0.883
**Previous stroke**	0.602	0.275–1.321	0.206			
**Previous PCI**	0.391	0.136–1.125	0.082	#	#	0.128
**Previous MI**	0.447	0.119–1.679	0.233			
**NLR**	0.970	0.917–1.026	0.289			
Monocyte (*10^**9**^**/L)**	0.624	0.204–1.910	0.409			
**Hemoglobin (g/L)**	1.034	1.019–1.049	< 0.001	#	#	0.165
Platelet (*10^**9**^**/L)**	1.000	0.996–1.004	0.852			
**TG (mmol/L)**	1.263	0.820–1.944	0.289			
**TC (mmol/L)**	1.074	0.820–1.408	0.604			
**LDL-C (mmol/L)**	0.994	0.722–1.370	0.973			
**HDL-C (mmol/L)**	2.753	0.813–9.324	0.104			
**VLDL-C (mmol/L)**	1.830	0.448–7.474	0.400			
**ApoA-I (g/L)**	4.694	1.147–19.205	0.031	6.189	1.169–32.763	0.032
**ApoB (g/L)**	0.624	0.201–1.931	0.413			
**Lp(a) (mg/L)**	0.995	0.993–0.996	< 0.001	0.996	0.994–0.997	< 0.001
eGFR (ml/(min*1.73m^**2**^**))**	1.059	1.042–1.075	< 0.001	1.053	1.035–1.071	< 0.001
**Uric acid (umol/L)**	0.994	0.992–0.997	< 0.001	#	#	0.212
**FBG (mmol/L)**	0.784	0.705–0.871	< 0.001	0.825	0.730–0.933	0.002

Values are presented with odds ratios and 95% confidence intervals. *P* < 0.05 indicated statistical significance

### The Lp(a) level, LVEF, and clinical endpoint events

The incidences of all-cause mortality and cardiac mortality were counted based on the Lp(a) subgroups (Table 4). Overall, 30 of 436 patients (6.9%) experienced all-cause mortality, and 26 of 436 patients (6.0%) had cardiac mortality. The incidence of all-cause mortality significantly differed between Tertile 1 and Tertile 2 and between Tertile 1 and Tertile 3.

**Table 4:** Mortality during follow-up

**Variables**	**Lp(a)**				**P Value**
	**Tertile 1**	**Tertile 2**	**Tertile 3**	**Total**	
**All-Cause Mortality,n (%)**	3 (2.1%)	13 (8.9%)	14 (9.7%)	30 (6.9%)	0.019
**Cardiac Mortality,n (%)**	3 (2.1%)	11 (7.5%)	12 (8.3%)	26 (6.0%)	0.051

Values are expressed as n (%). *P* values were calculated using the chi-square test or Fisher's test. *P* < 0.05 indicated statistical significance

Table 5 presents the univariate Cox regression analysis results of the independent variables as predictors for clinical endpoint events. The following significant and independent associations were identified (all *P* < 0.05): 1) hospitalization time, age, pulse pressure, DM, LVEF, NLR, monocyte, hemoglobin, ApoB, Lp(a), eGFR, uric acid, FBG with all-cause mortality; 2) hospitalization time, age, pulse pressure, LVEF, NLR, monocyte, hemoglobin, TG, ApoB, Lp(a), eGFR, uric acid, FBG with cardiac mortality.

**Table 5:** Univariate Cox regression analysis of all-cause mortality and cardiac mortality

**Variables**	**All-Cause Mortality**		**Cardiac Death**	
	**OR(95%CI)**	***P* value**	**OR(95%CI)**	***P* value**
**Hospitalization time**	0.719 (0.649–0.797)	< 0.001	0.672 (0.596–0.757)	< 0.001
**Age**	1.054 (1.021–1.089)	0.001	1.057 (1.021–1.094)	0.002
**Male**	0.533 (0.257–1.106)	0.091	0.484 (0.222–1.054)	0.068
**BMI**	0.939 (0.844–1.044)	0.243	0.948 (0.846–1.062)	0.357
**Heart rate**	1.009 (0.989–1.030)	0.361	1.014 (0.994–1.035)	0.182
**Pulse pressure**	0.939 (0.916–0.963)	< 0.001	0.933 (0.909–0.957)	< 0.001
**Current smoker**	1.004 (0.470–2.144)	0.993	0.890 (0.387–2.046)	0.783
**Current drinker**	0.776 (0.317–1.898)	0.578	0.735 (0.277–1.948)	0.535
**Hypertension**	1.353 (0.652–2.809)	0.417	1.438 (0.653–3.170)	0.367
**Diabetes mellitus**	2.218 (1.015–4.845)	0.046	2.291 (0.996–5.271)	0.051
**Previous stroke**	1.423 (0.545–3.721)	0.471	0.937 (0.281–3.123)	0.916
**Previous PCI**	0.747 (0.101–5.498)	0.774	0.046 (0.000–178.419)	0.466
**Previous MI**	1.207 (0.164–8.874)	0.854	0.048 (0.000–1464.702)	0.564
**LVEF**	0.922 (0.886–0.959)	< 0.001	0.921 (0.884–0.961)	< 0.001
**NLR**	1.126 (1.074–1.180)	< 0.001	1.124 (1.068–1.182)	< 0.001
**Monocyte**	8.519 (2.755–26.347)	< 0.001	10.423 (3.190–34.053)	< 0.001
**Hemoglobin**	0.972 (0.955–0.988)	0.001	0.968 (0.950–0.985)	< 0.001
**Platelet**	0.994 (0.988–1.000)	0.059	0.995 (0.988–1.001)	0.113
**TG**	0.596 (0.320–1.111)	0.103	0.475 (0.228–0.989)	0.047
**TC**	1.034 (0.744–1.438)	0.841	0.928 (0.644–1.337)	0.689
**LDL-C**	1.087 (0.735–1.608)	0.676	0.954 (0.615–1.479)	0.834
**HDL-C**	1.621 (0.430–6.121)	0.476	1.139 (0.258–5.030)	0.863
**VLDL-C**	0.198 (0.028–1.386)	0.103	0.231 (0.029–1.824)	0.164
**ApoA-I**	0.479 (0.083–2.770)	0.411	0.676 (0.106–4.308)	0.678
**ApoB**	4.760 (1.590–14.247)	0.005	4.256 (1.263–14.336)	0.019
**Lp(a)**	1.002 (1.001–1.004)	0.005	1.002 (1.001–1.004)	0.009
**eGFR**	0.960 (0.946–0.975)	< 0.001	0.959 (0.944–0.975)	< 0.001
**Uric acid**	1.004 (1.000–1.007)	0.027	1.004 (1.001–1.007)	0.024
**FBG**	1.218 (1.102–1.346)	< 0.001	1.243 (1.123–1.375)	< 0.001

Values are presented with odds ratios and 95% confidence intervals. *P* < 0.05 indicated statistical significance

The multivariate COX regression analysis using the stepwise forward method identified the following significant associations (all *P* < 0.05; Table 6): 1) hospitalization time, pulse pressure, LVEF, NLR, eGFR with all-cause mortality; 2) hospitalization time, pulse pressure, LVEF, NLR, eGFR with cardiac mortality. There was no association between the Lp(a) level and all-cause mortality and cardiac mortality (*P* = 0.133; *P* = 0.158). The best predictive Lp(a) for all-cause mortality (additional file 1. A) and cardiac mortality (additional file 1. B) by ROC curve analysis was 274.5 mg/L (accuracy: 0.6660, 95% CI: 0.5696–0.7623, sensitivity: 80.0%, and specificity: 55.7%, *P* = 0.0024, additional file 1. A; accuracy: 0.6499, 95% CI: 0.5423–0.7574, sensitivity: 76.9%, and specificity: 55.1%, *P* = 0.0103, additional file 1.B).

**Table 6:** Multivariate Cox regression analysis of mortality

**Variables**	**All-Cause Mortality**		**Cardiac Death**	
	**OR(95%CI)**	***P* value**	**OR(95%CI)**	***P* value**
**Hospitalization time**	0.760 (0.697–0.829)	< 0.001	0.728 (0.659–0.805)	< 0.001
**Age**	#	0.993	#	0.863
**Male**	#	0.477	#	0.408
**Pulse pressure**	0.935 (0.907–0.964)	< 0.001	0.924 (0.893–0.956)	< 0.001
**Diabetes mellitus**	#	0.790	#	0.682
**LVEF**	0.902 (0.848–0.959)	0.001	0.893 (0.834–0.956)	0.001
**NLR**	1.108 (1.049–1.172)	< 0.001	1.115 (1.048–1.186)	0.001
**Monocyte**	#	0.512	#	0.615
**Hemoglobin**	#	0.335	#	0.239
**Platelet**	#	0.930		0.699
**TG**	#	0.378	#	0.175
**ApoB**	#	0.993	#	0.687
**Lp(a)**	#	0.133	#	0.158
**eGFR**	0.977 (0.960–0.993)	0.006	0.977 (0.959–0.996)	0.016
**Uric acid**	#	0.279	#	0.301
**FBG**	#	0.634	#	0.790

Values are presented with odds ratios and 95% confidence intervals. *P* < 0.05 indicated statistical significance

**Fig. 3:**
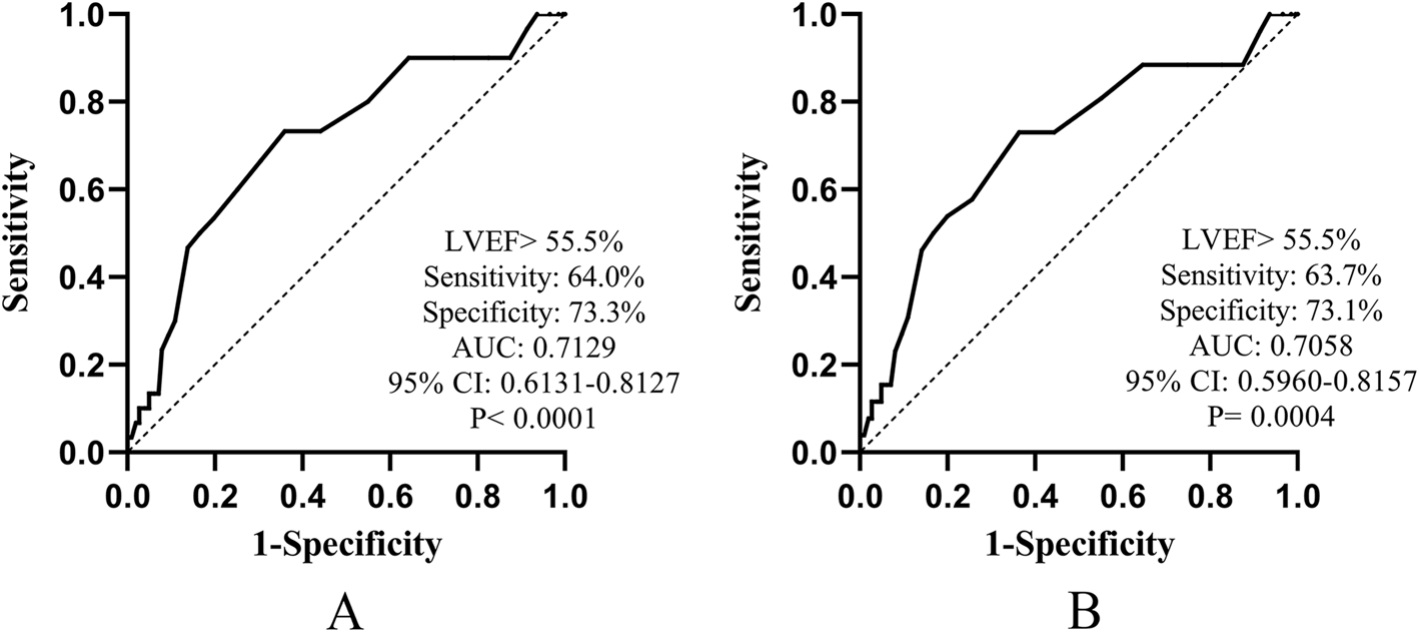
ROC curve analysis of LVEF for mortality. An LVEF cutoff value of 55.5% on admission predicted all-cause mortality (Fig. 3. A) and cardiac mortality (Fig. 3. B) in patients (accuracy: 0.7129, 95% CI: 0.6131–0.8127, sensitivity: 64.0%, and specificity: 73.3%, *P* < 0.0001, Fig. 3. A; accuracy: 0.7058, 95% CI: 0.5960–0.8157, sensitivity: 63.7%, and specificity: 73.1%, *P* = 0.0004, Fig. 3.B)

**Fig. 4:**
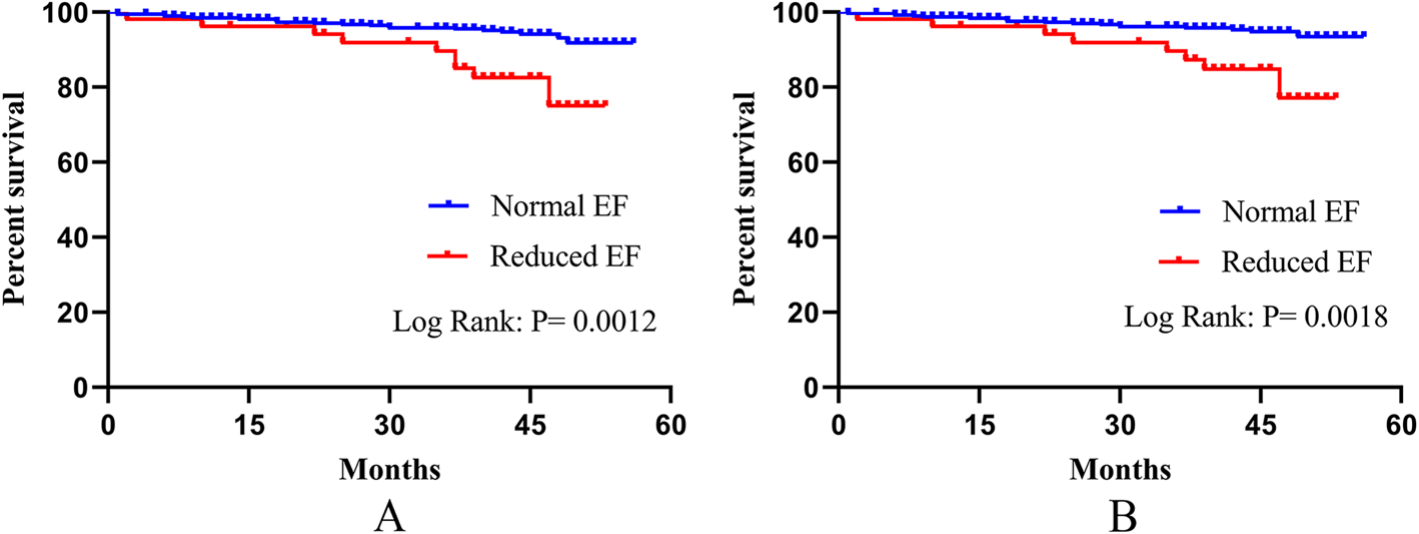
Kaplan‒Meier curves for all-cause mortality (Fig. 4. A) and cardiac mortality (Fig. 4. B) among reduced EF and normal EF

The best predictive LVEF for all-cause mortality and cardiac mortality by ROC curve analysis was 55.5% (accuracy: 0.7129, 95% CI: 0.6131–0.8127, sensitivity: 64.0%, and specificity: 73.3%, *P* < 0.0001, Fig. 3A; accuracy: 0.7058, 95% CI: 0.5960–0.8157, sensitivity: 63.7%, and specificity: 73.1%, *P* = 0.0004, Fig. 3B). The Kaplan–Meier analysis based on event-free survival indicated that the incidence of all-cause mortality and cardiac mortality decreased as LVEF increased (log-rank test; *P* = 0.0012, *P* = 0.0018; Fig. 4).

## Discussion

To our knowledge, this is a study to examine the correlation between the Lp(a) level and the LVEF, as well as the effect of both factors on mortality in patients with MI. We identified a significant negative correlation between the Lp(a) level and LVEF in patients with MI in China. Furthermore, Lp(a) levels > 455 mg/L could predict a reduced ejection fraction. However, the Lp(a) level did not affect mortality. In contrast, LVEF affected the incidence of all-cause mortality and cardiac mortality. Specifically, LVEF > 55.5% was the best predictive factor for all-cause mortality and cardiac mortality.

The Lp(a) particle is spherical in shape and 23.5–26.0 nm in diameter. Lp(a) is genetically regulated, mainly synthesized in the liver, and consists of lipids and proteins, with the lipid fraction being hydrophobic in the core and the periphery encapsulated in a protein complex composed of ApoB-100 and ApoA. Lp(a) concentrations vary greatly between individuals and races but remain stable throughout the individual’s lifetime, with minimal effects of gender, diet and environment. Lp(a) concentrations remain stable throughout life, with minimal effects of sex, diet and environment on Lp(a) [1]. Pathological mechanism of Lp(a) causing atherosclerosis (AS): 1, inhibition of fibrinolytic system: ApoA and fibrinogen have high structural homology with each other, Lp(a) competes with fibrinogen for fibrin binding sites and prevents the production of fibrin; Lp(a) prevents tissue fibrinolytic zymogen activator from binding to fibrin, making fibrinolytic zymogen unable to be activator of fibrinogen is activated into fibrinolytic enzyme; 2, promote the formation of foam cells: vascular endothelial cells are the main target cells of AS, Lp(a) can disrupt receptor-mediated endothelial diastolic function, leading to endothelial dysfunction; Lp(a) may be taken up by macrophages through receptor pathway and nonreceptor pathway, resulting in intracellular cholesterol accumulation into foam cells; Lp(a) can make platelets protein kinase-e substrate phosphorylation, increase platelet protein kinase-e activity, activate platelets and promote the formation of AS plaques; Lp(a) inhibits transforming growth factor-β1 and stimulates smooth muscle cell proliferation [1, 18]. Therefore, Lp(a) promotes atherosclerosis and thrombosis, which affects the hemodynamics of coronary arteries, decreases the blood supply to cells, and may lead to cell degeneration or even death in severe cases, which in turn leads to deterioration of cardiac function.

The Lp(a) concentration in the elderly group of patients with MI included in this study was greater than that in the younger age group, and we believe that because Lp(a) itself or its action by some related enzymes forms some small fragments that are then excreted by the kidneys, the poorer renal function of the elderly leads to elevated Lp(a) [1].

Aksoy, Mdeng et al. confirmed that high lipoprotein(a) levels may prolong occlusion of the culprit vessel and lead to greater myocardial necrosis and lower LVEF [19]. However, they did not further investigate Lp(a) for adverse prognostic events such as all-cause mortality and cardiac mortality. This study also confirmed that Lp(a) was associated with reduced LVEF in patients with MI, and after adjusting for confounders, there was still a moderately strong and independent association. We suggest two explanations for this: first, through proatherogenic and prothrombotic effects, elevated Lp(a) may lead to coronary thrombosis, which in turn impairs cardiac perfusion. This suggests that the relationship between Lp(a) and reduced ejection fraction is partially explained by reduced myocardial perfusion. Second, there is growing evidence that Lp(a) is an independent risk factor for aortic stenosis. Notably, aortic stenosis leads to a chronic elevation of left ventricular afterload, which is associated with cardiac necrosis and fibrosis, which in turn leads to a reduction in ejection fraction. This suggests that the relationship between Lp(a) and reduced ejection fraction is partly explained by aortic stenosis [20–22].

Multifactorial COX regression analysis suggested that Lp(a) was not a predictor of mortality, and we believe the reasons for this are the following: 1, Studies have found drugs and methods to reduce Lp(a) concentrations, including niacin, neomycin, lipoprotein apheresis, and antisense therapy targeting apolipoprotein(a) [23], but these methods were not routinely used for elevated Lp(a) in this study. In patients with dyslipidemia or unstable plaque, we routinely administered statins. While this decreases the incidence of adverse events, some cases have reported that statins may increase serum concentrations of Lp(a) [24]. Furthermore, this study lacks long-term Lp(a) concentration data since the patients were not assessed after discharge. This may affect the impact of Lp(a) on mortality. 2, Although the prognostic impact of Lp(a) on the Chinese population has not been determined, studies in other populations, such as white European patients [25], multicenter studies of patients in the United States and Canada [26], and Japanese patients [27], have reported adverse prognostic effects of Lp(a). Furthermore, an observational study of 460,506 participants (median follow-up: 11.2 years) reported significant differences in Lp(a) concentrations between races and populations (e.g., whites, South Asians, blacks, and Chinese) with differential effects of Lp(a) on cardiovascular disease [28]. Therefore, we speculate that our result could also be due to differences in the concentration and effects of Lp(a) among populations and races. 3, The American Heart Association published a statement recommending that Lp(a) be measured using an isomer-insensitive method in units of nmol/L. We measured Lp(a) in mg/L, which may have overestimated or underestimated the actual Lp(a) concentration. Therefore, our results might be related to the Lp(a) measurement method. One study used ApoA-independent measures to obtain Lp(a) concentrations, reporting that the Lp(a) level was a useful predictor of coronary heart disease [29].

A widely known fact is that a decrease in LVEF following a MI is a powerful indicator of poor prognosis [30]. In this study, multivariate Cox regression analysis suggested an independent and significant effect of LVEF on all-cause death and cardiogenic death. Normal ranges for LVEF as per the American Society of Echocardiography and the European Association of Cardiovascular Imaging are: LVEF (%) among the male population: 52% to 72% normal range。LVEF (%) among the female population:54% to 74% normal range. The best predictive value given by the ROC curve in this study regarding LVEF to predict all-cause mortality and cardiac mortality was 55.5%, which is close to and above the lower limit of normal ejection fraction for both male and female populations.

Current therapies to decrease Lp(a) include niacin, neomycin, Lp(a) monolectomy and antisense therapy targeting apolipoprotein(a). However, no benefit in reducing the risk of cardiovascular disease was observed when niacin was added to statins, and in addition, severe adverse reactions were observed [31]. Neomycin is an aminoglycoside broad-spectrum antibiotic that works well against gram-negative and gram-positive bacteria and *Mycobacterium tuberculosis*. Neomycin side effects are mainly gastrointestinal reactions, including loss of appetite and nausea. In addition, comparable to similar antibiotics, it has nephrotoxicity and inner ear toxicity, and it causes damage to the inner ear, often irreversibly. Therefore, in routine clinical practice, neomycin is used more for anti-infection than for lowering Lp(a). Lipid apheresis is a nonsurgical therapy that removes high LDL-C and Lp(a) from the blood. Lipid apheresis is a two- to three-hour procedure where a person is connected to a special machine that filters their blood. The plasma portion of the blood, which contains cholesterol, is separated and run through the machine to remove LDL and Lp(a) before the blood is returned to the body. While encouragingly, Georgiana-Aura Giurgea et al. found that regular and long-term lipid apheresis in patients with familial hypercholesterolemia (FH) significantly increased LVEF independent of statin therapy [32]. Meanwhile, the new promising antisense oligonucleotides can bind to hepatic LPA mRNA and reduce Lp(a), but they still need to be further tested [33].

We believe that elevated Lp(a) implies reduced LVEF in patients with MI, and LVEF, but not Lp(a), can impact long-term mortality. This conclusion still needs to be further evaluated in larger studies.

### Study strengths and limitation

This is the first study to examine the correlation between Lp(a) level and LVEF in Chinese patients with myocardial infarction and the effect of Lp(a) level and LVEF on long-term mortality. However, there are some limitations in this study: First, this was an observational study susceptible to confounding factors that may have affected our findings. Second, this was a single-center study with an inadequate sample size, leading to selection bias; a prospective, multicenter study with a larger sample size is needed to confirm these findings. Third, we tried to collect information during the implementation phase of the study to avoid lost follow-up, such as cell phone numbers and WeChat. of the patients and their relatives and establish a good relationship with them. However, it was still impossible to avoid missing follow-ups. Eleven patients were lost in this study, accounting for less than 3% of the total population. This may produce bias, which is a limitation of this study. Finally, we only reported the patients’ baseline characteristics at hospitalization, and long-term laboratory findings after discharge were lacking. Thus, continuous dynamic measurements would increase the accuracy of the results.

## Conclusion

The Lp(a) concentration and LVEF were significantly and negatively correlated (r = -0.407, β = -0.349, *P* < 0.001) in patients with MI. Furthermore, an Lp(a) concentration of > 455 mg/L was a predictive factor for reduced LVEF. However, the Lp(a) concentration did not affect mortality. In contrast, LVEF significantly affected all-cause mortality and cardiac mortality; LVEF over 55.5% had the best predictive abilities. Overall, our results suggest that an elevated Lp(a) concentration predicts reduced LVEF and that LVEF predicts mortality in patients with MI.

## Supplementary Information

**Additional file 1.** ROC curve analysis of Lp(a) for mortality. Lp(a) cutoff value of 274.5 mg/L on admission predicts all-cause mortality (additional file 1. A) and cardiac mortality (additional file 1. B) in patients (accuracy: 0.6660, 95% CI: 0.5696–0.7623, sensitivity: 80.0%, and specificity: 55.7%, P =0.0024, additional file 1. A; accuracy: 0.6499, 95% CI: 0.5423–0.7574, sensitivity: 76.9%, and specificity: 55.1%, P =0.0103, additional file 1.B).

## Data Availability

The datasets analyzed during the current study are not publicly available due to privacy protection but are available from the corresponding author on reasonable request.

## Abbreviations

Lp(a): Lipoprotein (a)
LVEF: Left ventricular ejection fraction
MI: Myocardial infarction
NSTEMI: Non-ST-segment elevation myocardial infarction
STEMI: ST-elevation myocardial infarction
ROC: Receiver operating characteristic curve
AUC: Area under the curve
PCI: Percutaneous coronary intervention
CABG: Coronary artery bypass grafting
SYNTAX: Score, synergy between percutaneous coronary intervention with taxus and cardiac surgery score
BMI: Body mass index
DM: Diabetes mellitus
TG: Triglycerides
TC: Total cholesterol
LDL-C: Low-density lipoprotein cholesterol
HDL-C: High-density lipoprotein cholesterol
VLDL-C: Very low-density lipoprotein cholesterol
ApoA-I: Apolipoprotein A-I
ApoB: Apolipoprotein B
eGFR: Glomerular filtration rate
FBG: Fasting blood glucose
NLR: Neutrophil-to-lymphocyte ratio
