# Supplementary figures and images for: Lipoprotein (a) and myocardial infarction: impact on long-term mortality
Source: Lipids Health Dis. 2023 Jun 9;22:70. doi: 10.1186/s12944-023-01841-z (PMC10251652; doi:10.1186/s12944-023-01841-z)

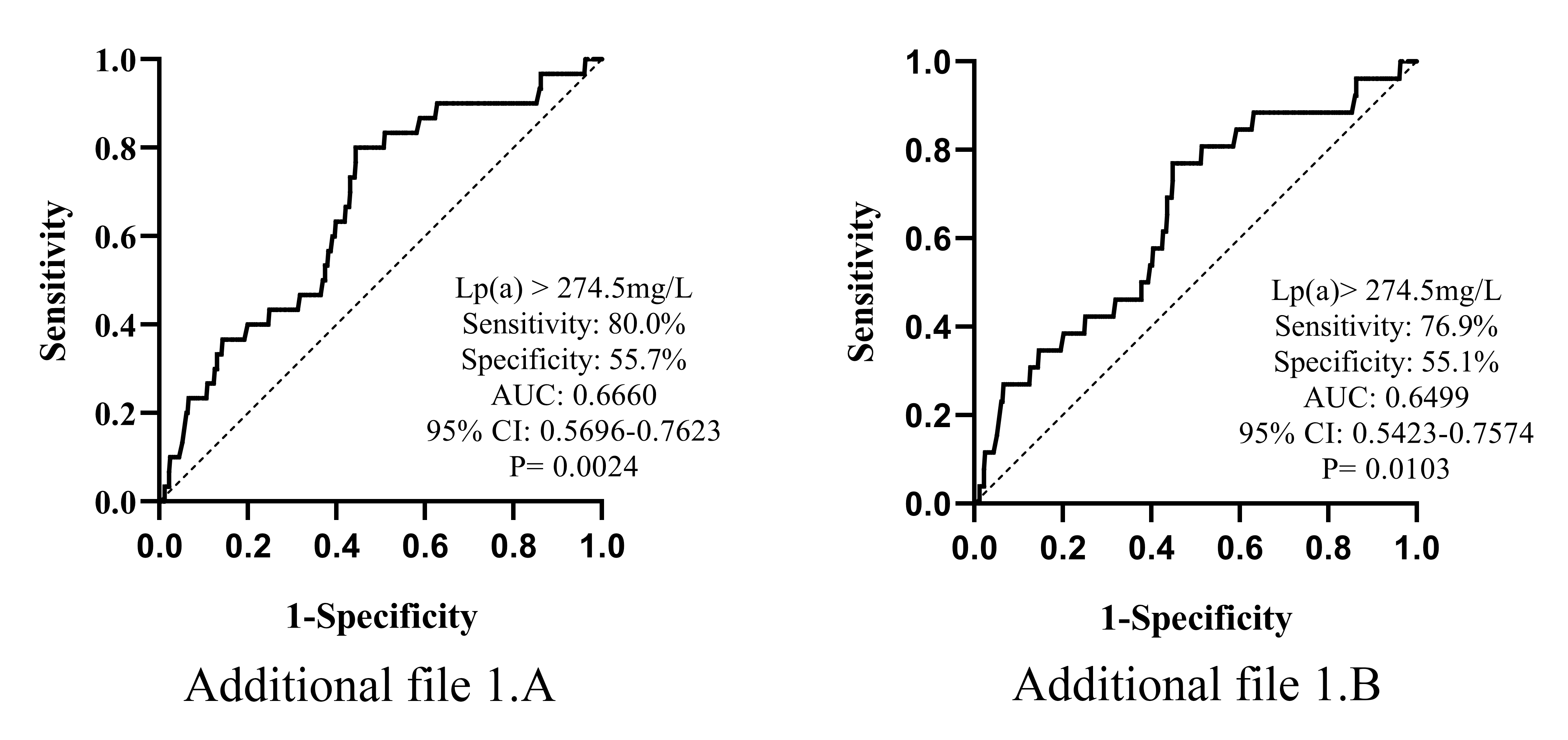

Supplement: Supplementary file 1 — Additional file 1. ROC curve analysis of Lp(a) for mortality. Lp(a) cutoff value of 274.5 mg/L on admission predicts all-cause mortality (additional file 1. A) and cardiac mortality (additional file 1. B) in patients (accuracy: 0.6660, 95% CI: 0.5696–0.7623, sensitivity: 80.0%, and specificity: 55.7%, P =0.0024, additional file 1. A; accuracy: 0.6499, 95% CI: 0.5423–0.7574, sensitivity: 76.9%, and specificity: 55.1%, P =0.0103, additional file 1.B). [file 12944_2023_1841_MOESM1_ESM.tif]
